# Role of muscle ultrasound in frailty assessment in older adults with type 2 diabetes mellitus

**DOI:** 10.1186/s12877-024-05008-y

**Published:** 2024-05-04

**Authors:** Merve Hafızoğlu, Hatice Kübra Yıldırım, Arzu Okyar Baş, Didem Karaduman, Zeynep Şahiner, Burcu Balam Doğu, Meltem Gülhan Halil, Mustafa Cankurtaran, Cafer Balcı

**Affiliations:** 1 Department of Internal Medicine, Division of Geriatrics, Hacettepe University Faculty of Medicine, Ankara, Turkey; 2 Department of Internal Medicine, Hacettepe University Faculty of Medicine, Ankara, Turkey

**Keywords:** Frailty, Muscle ultrasound, Older adults, Diabetes mellitus

## Abstract

**Background:**

Frailty is a geriatric syndrome that is characterized by increased vulnerability to intrinsic and extrinsic stressors due to decreased biologic reserves. Muscle ultrasound (US) is a valid and reliable method for assessing muscle quantity in older adults. The study aims to examine the relationship between frailty definitions and US-derived muscle parameters.

**Methods:**

We conducted a cross-sectional study with type 2 diabetes mellitus outpatients in a tertiary hospital, and all participants underwent a comprehensive geriatric assessment. For frailty assessment, the Fried Frailty Phenotype (FFP), the Clinical Frailty Scale (CFS), and the Edmonton Frailty Scale (EFS) were performed. Muscle US measurements included Gastrocnemius Medialis (GM) muscle thickness, GM fascicle length, GM pennation angle, Rectus Femoris (RF) muscle thickness, Rectus Femoris cross-sectional area (RFCSA), Rectus Abdominis (RA) muscle thickness, External Oblique (EO) muscle thickness, Internal Oblique (IO) muscle thickness, and Transverse Abdominis (TA) muscle thickness.

**Results:**

In all, 373 participants were included in the study. The median age of participants was 72.7 ± 5.9 years, and 64.6% of them were female. According to the FFP, 18.2% of the participants were living with frailty, 56% of them were pre-frail; 57.4% of them were living with frailty according to the CFS; 25.2% of them were living with frailty, and 20.6% of them were pre-frail according to the EFS. The FFP, CFS, and EFS scores were related to muscle thickness of GM, RF, and RA, fascicle length of GM, and pennation angle of GM and RFCSA. Particularly, GM pennation angle, RF muscle thickness, and RFCSA were associated with an increased risk of frailty. Besides muscle thickness of GM, RF, and RA, fascicle length of GM, pennation angle of GM, and RFCSA were significant for predicting the presence of frailty.

**Conclusions:**

US-derived regional muscle measurements are associated with frailty definitions (in both physical, cumulative deficit, and multidimensional models) in a diabetic geriatric population.

**Supplementary Information:**

The online version contains supplementary material available at 10.1186/s12877-024-05008-y.

## Background

Frailty is a condition that is characterized by increased susceptibility to intrinsic and extrinsic stressors due to decreased biologic reserves, and it is associated with adverse outcomes such as mortality, hospitalization, falls, and admission to long-term care. It can be considered a geriatric syndrome and can also be seen at any age due to chronic diseases. Its multidimensional structure is formed by physical, psychological, biological, nutritional, sociodemographic, and environmental factors. Since it is a dynamic process, it can be prevented when detected in the reversible stage or progress rapidly if not intervened [1]. Two models have been determined for frailty assessment: the phenotype model and the cumulative deficit model. While the phenotype model mostly detects frailty based on a physical condition, such as the Fried Frailty Phenotype (FFP), the cumulative model evaluates frailty as a continuous state, taking into account the functional decline and disability caused by the current situation, such as the Clinical Frailty Scale (CFS) and the Edmonton Frailty Scale (EFS) [2, 3]. It has been shown in many different populations that individuals living with frailty have a poor prognosis and cause much more healthcare costs, regardless of which method is performed. Although the current prevalence varies in the range of 4–59%, considering the increase in the older population all over the world, the detection and prevention of frailty have become very crucial [4, 5].

Muscle evaluation with ultrasound (US) is a current issue, and it provides superiority over gold standard methods such as computed tomography (CT), magnetic resonance imaging (MR), dual-energy x-ray absorptiometry (DXA), and bioelectrical impedance analysis (BIA) due to its low cost, easy application, and radiation-free nature [6]. Furthermore, studies have demonstrated its correlation with measurements obtained through CT and BIA [7]. It is valid and reliable for muscle measurements and also for older adults. Because of these clinical properties, the European Geriatric Medicine Society sarcopenia group published a report for standardization of US measurements to assess muscle mass [8]. Previous studies have shown that muscle measurements obtained by the US are associated with adverse outcomes in intensive care patients, worse overall survival, and postoperative complications in cancer patients [9–11]. At the same time, studies on frailty have shown the relationship between US measurements and frailty in hemodialysis patients [12]. And a pilot study showed that thigh muscle thickness derived from US was associated with sarcopenia in older frail patients with type 2 diabetes mellitus [13]. However, the aforementioned studies focused on a single muscle group, which is usually a part of the anterior thigh muscles, and a single scale was generally used for the diagnosis of frailty. And most studies were conducted with patients in hospital or long-term nursing home or in need of intensive care.

The study aims to examine the relationship of US-derived muscle thickness, fascicle length, pennation angle, and cross-sectional area of more than one muscle group with the Fried Frailty Phenotype (FFP), Clinical Frailty Scale (CFS), and Edmonton Frailty Scale (EFS) in community-dwelling older adults with type 2 diabetes mellitus (DM).

## Methods

### Study population

This was a cross-sectional study conducted from October to December 2023 in a tertiary hospital. Type 2 DM outpatients who were able to cooperate and orient in comprehensive geriatric assessment tests were included. Demographic data including age, sex, and DM characteristics such as DM duration, treatments, complications, and HbA1c levels were recorded. Geriatric syndromes such as dementia, depression, osteoporosis, urinary incontinence, fall history and frailty were noted for each patient. Three different definitions were used for the frailty status. Muscle ultrasound was performed to all participants. Patients with advanced dementia, decompensated heart failure, end-stage kidney and liver disease, end-stage cancer, and those with severe vision and hearing problems were excluded from the study. STROBE Statement-Checklist were controlled for reporting this study.

### Comprehensive geriatric assessment

For Comprehensive Geriatric Assessment (CGA); Katz Activities of Daily Living (ADL), Lawton-Brody Instrumental Activities of Daily Living (IADL), Mini-Mental State Examination (MMSE), Mini-Nutritional Assessment-short form (MNA-sf), SARC-f, and Yesavage Geriatric Depression Scale (YGDS) were performed.

The Katz-ADL is a scale in which bathing, dressing, toileting, transferring, continency, and feeding are questioned; each scored 1 point, and increasing scores are associated with independence. A validity and reliability study is available in Turkish [14, 15]. The Lawton-Brody IADL is a scale consisting of the ability to use the telephone, shop, food preparation, housekeeping, laundry, mode of transportation, responsibility for own medications, and ability to handle finances categories, where each category is scored as 0 or 1, and increasing scores indicate independency. There is a Turkish validity and reliability study [16, 17]. The MMSE is a cognitive disorder screening test that evaluates orientation, attention/concentration, recall, language skills, visuospatial abilities, and the ability to understand and follow instructions. The maximum score is 30, and a score below 24 indicates possible cognitive impairment [18, 19]. The MNA-sf is a practical screening tool for malnutrition and malnutrition risk. Food intake over the past three months, weight loss during the last 3 months, mobility, psychological stress or acute illness in the past three months, neuropsychological problems, and body mass index (BMI) are questioned. 0–7 points are considered malnutrition, and 8–11 points are malnutrition risk [20, 21]. The SARC-f questionnaire is a screening tool for identifying probable sarcopenic patients. Difficulty in lifting 5 kg, assistance in walking, difficulty in transferring from a chair, difficulty in climbing a flight of 10 stairs, and fall history in the past year are questioned. Each question is scored from 0 to 2, with the greatest maximum score of 10, and scores of 4 and above indicate the risk of sarcopenia [22]. The YGDS is a 15-item instrument designed to screen depression probability in geriatric populations, and items require a yes/no response. Values of 5 and above indicate the risk of depression [23, 24]. All tests have Turkish reliability and validity studies; Turkish versions of all tests were used in the study.

### Frailty assessment

For frailty assessment, the Fried Frailty Phenotype (FFP), the Clinical Frailty Scale (CFS), and the Edmonton Frailty Scale (EFS) were performed.

The FFP is a commonly used tool for detecting physical frailty, and it includes five criteria: unintentional weight loss; weakness or poor handgrip strength; self-reported exhaustion; slow walking speed; and low physical activity. A total score of 0 means that a person is robust or not frail; 1–2 prefrail; and 3 and above means frail [2, 25]. The CFS is a judgment-based tool to screen for frailty. An individual’s frailty status is scored from 0 to 9 with the aid of a visual chart by an experienced clinician. Level 1 indicates very fit; level 2 fit; level 3 managing well; level 4 living with very mild frailty; level 5 living with mild frailty; level 6 living with moderate frailty; level 7 living with severe frailty; level 8 living with very severe frailty; and level 9 terminally ill [26, 27]. The EFS is a valid and reliable frailty detection tool consisting of 9 items (cognition, general health status, functional independence, social support, medication use, nutrition, mood, continuity, and functional performance). Total score from 0 to 5 points indicates robust; 6–7 points, apparently vulnerable; 8–9 points, mildly frail; 10–11 points, moderately frail; 12–17 points indicate severe frailty [28, 29].

### Muscle ultrasound

Ultrasound measurements (Gastrocnemius Medialis **(**GM) muscle thickness, GM fascicle length, GM pennation angle, Rectus Femoris (RF) muscle thickness, Rectus Femoris cross-sectional area (RFCSA), Rectus Abdominis **(**RA) muscle thickness, External Oblique (EO) muscle thickness, Internal Oblique (IO) muscle thickness, Transverse Abdominis (TA) muscle thickness) were performed using a 10 MHz linear probe of 5 cm width ((LOGIQ200pro) General Electrics Medical Systems)). Water-soluble transmission gel was used to reduce pressure and provide acoustic contact. All measurements were performed by the same physician. For muscle thickness measurement, transversal images of the distance were captured between the superficial and the deep fascia. GM muscle measurements were performed from the proximal 30% point between the medial point of the articular cleft of the knee and the medial top of the medial malleolus in a sitting position. RF muscle measurements were performed from the midpoint of the anterior superior iliac spine and superior of the patella. The images of abdomen muscles were captured at the end of the expirium from the right side of the body, 3 cm lateral to the umbilicus. For fascicle length measurement, the distance between the insertions of the fascicle into the superficial and deep aponeuroses was measured. For pennation angle measurement, the probe rotated, and the longitudinal view was captured, the distance between muscle fibers and the deep fascia of the muscle was measured. For RFCSA measurement, the area of the cross-section of a muscle perpendicular to its longitudinal axis was measured. All measurements were taken twice, and the mean values were used for analysis. For assessment of intraobserver reliability, intraclass correlation coefficients (ICC) were evaluated using two images taken 20 min intervals of 20 healthy participants. The ICCs were 0.96, 0.96, and 0.94 for muscle thickness of the GM, RF, and RA respectively; were 0.98 for RFCSA.

### Statistical analyses

Statistical analyses were performed using the SPSS software version 25. The variables were investigated using histograms, probability plots, and analytic methods (Kolmogorov-Simirnov/Shapiro-Wilk’s test) to determine whether or not they are normally distributed. Descriptive analyses were presented using mean ± standard deviation for the normally distributed variables; median (min-max) for the non-normally distributed variables. Since frailty index scores were not normally distributed, the correlation coefficients and their significance were calculated using the Spearman test. Binary logistic regression analyses were performed on both unadjusted and adjusted models. Covariables were chosen from proven or clinically suspected risk factors with frailty (Model-1: Unadjusted model, Model-2: Sex adjusted model, Model-3: Age, sex adjusted model, Model-4: Age, sex, BMI adjusted model). The capacity of muscle measurements in predicting the presence of frailty was analyzed using ROC (Receiver Operating Characteristics) curve analysis. A 5% type 1 error level was used to define statistical significance. The total sample size was determined by G-power analysis. When effect size = 0.30 α = 0.05, and power (1-ß) = 0.99 was accepted, the total sample size was calculated as 356. The effect size value of 0.30 utilized in the analysis was determined based on previous literature in the field, which reported effect sizes within a similar range for the relationship under investigation [12].

## Results

**Fig. 1:**
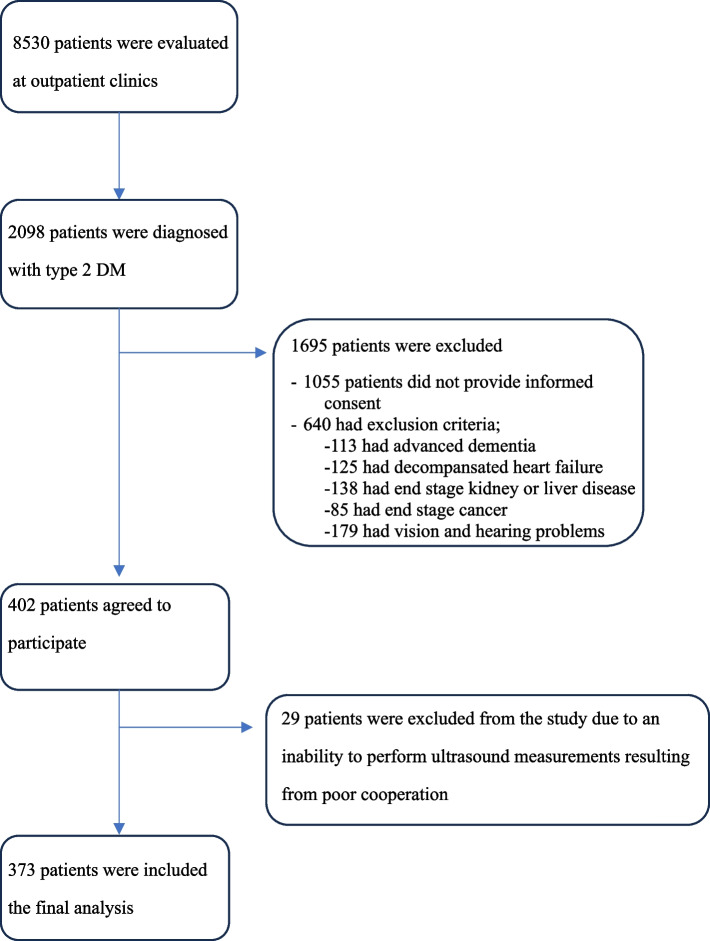
Flowchart of study population

In all, 373 patients with type 2 DM who admitted to geriatrics outpatient clinic were enrolled. The flowchart of the study population was presented in Fig. 1. The median age of participants was 72.7 ± 5.9 years (ranges between 65 and 90), and 64.6% of them were female. The median HbA1c level was 7.2% (4.9–18.3). The baseline characteristics of the participants were given in Table 1.

**Table 1.** Baseline characteristics of participants

		* N* =373
Age (year) (mean±SD (min-max))		72.7±5.9 (65-90)
Female gender (n (%))		241 (64.6%)
**DM characteristics**		
DM duration (year)		10 (1-40)
DM treatment	OAD (n (%))	224 (60%)
	Insulin (n (%))	22 (5.9%)
	OAD+Insulin (n (%))	106 (28.4%)
DM complication	Nephropathy (n (%))	77 (20.6%)
	Neuropathy (n (%))	165 (44.2%)
	Retinopathy (n (%))	48 (12.9%)
	CAD (n (%))	128 (34.3%)
	CVD (n (%))	40 (10.7%)
HbA1c (%) (median (min-max))		7.2 (4.9-18.3)
BMI (kg/m²) (mean±SD)		31±5.4
**Comprehensive Geriatric Assessment**		
ADL (median (min-max))		6 (0-6)
IADL (median (min-max))		8 (0-8)
MMSE (median (min-max))		28 (7-30)
MNA-sf (median (min-max))		13 (0-14)
SARC-f (median (min-max))		1 (0-10)
YGDS (median (min-max))		2 (0-15)
**Geriatric Syndromes**		
Dementia (n (%))		30 (8%)
Depression (n (%))		22 (5.9%)
Osteoporosis (n (%))		48 (12.9%)
Urinary Incontinence (n (%))		160 (42.9%)
Fall history (n (%))		115 (30.8%)
**Frailty status according to Frailty tools**		
FFP	Robust (n (%))	96 (25.7%)
	Pre-frail (n (%))	209 (56%)
	Living with frailty (n (%))	68 (18.2%)
CFS	Robust (n (%))	159 (42.6%)
	Living with frailty (n (%))	214 (57.4%)
EFS	Robust (n (%))	200 (53.6%)
	Pre-frail (n (%))	77 (20.6%)
	Living with frailty (n (%))	94 (25.2%)
**Muscle Ultrasound**		
GM muscle thickness (mm) (mean±SD)		15.3±2.9
GM fascicle length (mm) (mean±SD)		28.8±5.2
GM pennation angle (°) (mean±SD)		24.8±5.9
RF muscle thickness (mm) (mean±SD)		14.3±3.5
RFCSA (cm²) (mean±SD) (median (min-max))		5.5±1.9
RA muscle thickness (mm) (mean±SD)		7.3±1.9
EO muscle thickness (mm) (median (min-max))		3.4 (1.3-8.9)
IO muscle thickness (mm) (mean±SD)		5.7±1.9
TA muscle thickness (mm) (median (min-max))		3.8 (1.4-8.8)

*DM* Diabetes Mellitus, *OAD* Oral antidiabetic, *CAD* Coronary Artery Disease, *CVD* Cerebrovascular Disease, *ADL* Activities of Daily Living, *IADL* Instrumental Activities of Daily living, *MMSE* Mini mental state examination, *MNA-sf* Mini Nutritional Assessment-short form, *YGDS* Yesevage Geriatric Depression Scale, *FFP* Fried Frailty Phenotype, *CFS* Clinical Frailty Scale, *EFS* Edmonton Frailty Scale, *GM* Gastrocnemius Medialis, *RF* Rectus Femoris, *RFCSA* Rectus Femoris cross sectional area, *RA* Rectus Abdominis, *EO* External Oblique, *IO* Internal Oblique, *TA* Transverse Abdominis

**Fig. 2:**
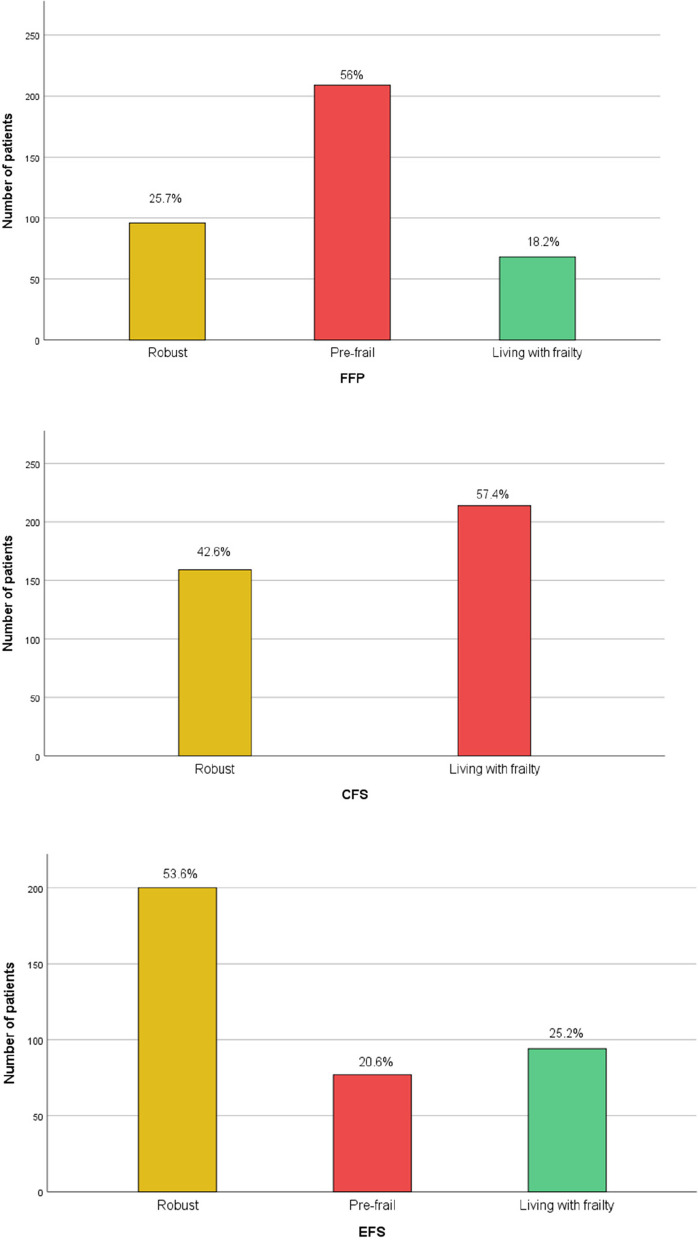
Bar-chart graphs of the frailty status of participants according to frailty definitions. (FFP: Fried Frailty Phenotype, CFS: Clinical Frailty Scale, EFS: Edmonton Frailty Scale)

In comprehensive geriatric assessments, the median ADL score was 6 [0–6], IADL score was 8 [0–8], MMSE score was 28 [7–30], MNA-sf score was 13 [0–14], SARC-f score was 1 [0–10], YGDS score was 2 [0–15]. Among geriatric syndromes, the prevalence of dementia was 8%, depression was 5.9%, osteoporosis was 12.9%, urinary incontinence was 42.9%, and fall was 30.8%. According to the FFP, 18.2% of the participants were living with frailty, 56% of them were prefrail, 57.4% of them were living with frailty according to the CFS, 25.2% of them were living with frailty, and 20.6% of them were prefrail according to the EFS (Fig. 2). The mean and median values of muscle measurements were given in Table 1.

Correlations between the FFP scores and GM muscle thickness, GM fascicle length, GM pennation angle, RF muscle thickness, RFCSA, RA muscle thickness, and IO muscle thickness were statistically significant (respectively, r-0.342, r-0.167, r-0.164, r-0.372, r-0.408, r-0.238, r-0.107). The CFS scores and GM muscle thickness, GM fascicle length, GM pennation angle, RF muscle thickness, RFCSA, and RA muscle thickness were significantly correlated (respectively, r-0.363, r-0.180, r-0.181, r-0.360, r-0.366, r-0.237). The EFS scores and GM muscle thickness, GM fascicle length, GM pennation angle, RF muscle thickness, RFCSA, RA muscle thickness, and IO muscle thickness were significantly correlated (respectively, r-0.396, r-0.251, r-0.204, r-0.323, r-0.371, r-0.257, r-0.126) (Table 2).

**Table 2:** Correlations between muscle measurements and frailty definitions

	GM muscle thickness	GM fascicle length	GM pennation angle	RF muscle thickness	RFCSA	RA muscle thickness	EO muscle thickness	IO muscle thickness	TA muscle thickness
FFP	**r: -0.342** *p* < 0.001	**r: -0.167** *p* = 0.001	**r: -0.164** *p* = 0.002	**r: -0.372** *p* < 0.001	**r: -0.408** *p* < 0.001	**r: -0.238** *p* < 0.001	r: -0.040 *p*: 0.446	**r: -0.107** *p*: 0.040	r: 0.015 *p*: 0.776
CFS	**r: -0.363** *p* < 0.001	**r: -0.180** *p* < 0.001	**r: -0.181** *p* < 0.001	**r: -0.360** *p* < 0.001	**r: -0.366** *p* < 0.001	**r: -0.237** *p* < 0.001	r: -0.033 *p*: 0.529	r: -0.088 *p*: 0.091	r: 0.049 *p*: 0.353
EFS	**r: -0.396** *p* < 0.001	**r: -0.251** *p* < 0.001	**r: -0.204** *p* < 0.001	**r: -0.323** *p* < 0.001	**r: -0.371** *p* < 0.001	**r: -0.257** *p* < 0.001	r: -0.083 *p*: 0.114	**r: -0.126** *p*: 0.016	r: 0.061 *p*: 0.243

*FFP* Fried Frailty Phenotype, *CFS* Clinical Frailty Scale, *EFS* Edmonton Frailty Scale, *GM* Gastrocnemius Medialis, *RF* Rectus Femoris, *RFCSA* Rectus Femoris cross sectional area, *RA* Rectus Abdominis, *EO* External Oblique, *IO* Internal Oblique, *TA*: Transverse Abdominis

Bold values indicate statistical significance

In univariate regression analyses, GM muscle thickness, GM pennation angle, RF muscle thickness, RFCSA, and RA muscle thickness were associated with the FFP, CFS, and EFS. GM fascicle length was only associated with the FFP and EFS scores. In age, sex, and BMI adjusted multivariate analyses, GM pennation angle (OR: 0.95, CI 95%: 0.91–0.99), RF muscle thickness (OR: 0.87, CI 95%: 0.80–0.95), RFCSA (OR: 0.78, CI 95%: 0.67–0.92) were associated with the FFP scores; GM pennation angle (OR: 0.95, CI 95%: 0.91–0.99), RF muscle thickness (OR: 0.88, CI 95%: 0.81–0.95), RFCSA (OR: 0.78, CI 95%: 0.67–0.91), RA muscle thickness (OR: 0.85, CI 95%: 0.74–0.98) were associated with the CFS scores; GM muscle thickness (OR: 0.88, CI 95%: 0.79–0.98), GM pennation angle (OR: 0.94, CI 95%: 0.89–0.98), RF muscle thickness (OR: 0.87, CI 95%: 0.80–0.96), RFCSA (OR: 0.76, CI 95%: 0.63–0.91) were associated with the EFS scores (Table 3).

**Table 3:** Logistic regression analyses results in showing the association between muscle measurements and frailty definitions

	FFP		CFS		EFS	
**Model-1**						
	OR (CI 95%)	*P* value	OR (CI 95%)	*P* value	OR (CI 95%)	*P* value
GM muscle thickness	0.83 (0.76–0.90)	**< 0.001**	0.83 (0.77–0.89)	**< 0.001**	0.78 (0.72–0.86)	**< 0.001**
GM fascicle length	0.95 (0.91–0.99)	**0.047**	0.96 (0.93-1.00)	0.123	0.91 (0.87–0.96)	**0.001**
GM pennation angle	0.93 (0.89–0.97)	**< 0.001**	0.94 (0.91–0.97)	**0.001**	0.92 (0.88–0.96)	**< 0.001**
RF muscle thickness	0.83 (0.77–0.89)	**< 0.001**	0.84 (0.78–0.89)	**< 0.001**	0.81 (0.75–0.87)	**< 0.001**
RFCSA	0.72 (0.63–0.81)	**< 0.001**	0.72 (0.64–0.81)	**< 0.001**	0.65 (0.56–0.76)	**< 0.001**
RA muscle thickness	0.80 (0.71–0.90)	**< 0.001**	0.82 (0.73–0.92)	**0.001**	0.76 (0.66–0.88)	**< 0.001**
**Model-2**						
GM muscle thickness	0.85 (0.78–0.93)	**0.001**	0.85 (0.78–0.92)	**< 0.001**	0.81 (0.74–0.89)	**< 0.001**
GM fascicle length	0.97 (0.92–1.01)	0.212	0.98 (0.94–1.02)	0.490	0.93 (0.89–0.98)	**0.015**
GM pennation angle	0.93 (0.90–0.97)	**0.002**	0.94 (0.91–0.98)	**0.003**	0.92 (0.88–0.96)	**0.001**
RF muscle thickness	0.83 (0.77–0.90)	**< 0.001**	0.85 (0.79–0.91)	**< 0.001**	0.83 (0.76–0.91)	**< 0.001**
RFCSA	0.72 (0.62–0.84)	**< 0.001**	0.73 (0.64–0.84)	**< 0.001**	0.69 (0.58–0.82)	**< 0.001**
RA muscle thickness	0.83 (0.73–0.96)	**0.01**	0.87 (0.77–0.98)	**0.028**	0.82 (0.70–0.96)	**0.017**
**Model-3**						
GM muscle thickness	0.94 (0.85–1.04)	0.306	0.95 (0.86–1.04)	0.274	0.89 (0.81–0.99)	**0.040**
GM fascicle length	0.99 (0.94–1.05)	0.899	1.02 (0.97–1.06)	0.380	0.97 (0.92–1.02)	0.284
GM pennation angle	0.95 (0.91–0.99)	**0.033**	0.96 (0.92-1.00)	0.076	0.94 (0.90–0.99)	**0.020**
RF muscle thickness	0.87 (0.80–0.95)	**0.002**	0.89 (0.82–0.96)	**0.005**	0.88 (0.80–0.96)	**0.008**
RFCSA	0.79 (0.67–0.92)	**0.003**	0.81 (0.70–0.93)	**0.005**	0.77 (0.64–0.92)	**0.005**
RA muscle thickness	0.87 (0.75–1.01)	0.068	0.92 (0.80–1.05)	0.221	0.88 (0.74–1.04)	0.140
**Model-4**						
GM muscle thickness	0.94 (0.85–1.04)	0.292	0.93 (0.85–1.02)	0.135	0.88 (0.79–0.98)	**0.024**
GM fascicle length	0.99 (0.94–1.04)	0.896	1.02 (0.97–1.07)	0.414	0.97 (0.92–1.02)	0.251
GM pennation angle	0.95 (0.91–0.99)	**0.027**	0.95 (0.91–0.99)	**0.015**	0.94 (0.89–0.98)	**0.012**
RF muscle thickness	0.87 (0.80–0.95)	**0.002**	0.88 (0.81–0.95)	**0.002**	0.87 (0.80–0.96)	**0.006**
RFCSA	0.78 (0.67–0.92)	**0.002**	0.78 (0.67–0.91)	**0.002**	0.76 (0.63–0.91)	**0.004**
RA muscle thickness	0.86 (0.75-1.00)	0.052	0.85 (0.74–0.98)	**0.031**	0.85 (0.71-1.00)	0.064

*FFP* Fried Frailty Phenotype, *CFS* Clinical Frailty Scale, *EFS* Edmonton Frailty Scale, *GM* Gastrocnemius Medialis, *RF* Rectus Femoris, *RFCSA* Rectus Femoris cross sectional area, *RA* Rectus Abdominis, *EO* External Oblique, *IO* Internal Oblique, *TA* Transverse Abdominis

Model-1. Univariate analyses, Model-2. Sex adjusted multivariable analyses, Model-3. Age, sex adjusted multivariable analyses, Model-4. Age, sex, BMI adjusted multivariable analyses.

Bold values indicate statistical significance

**Fig. 3:**
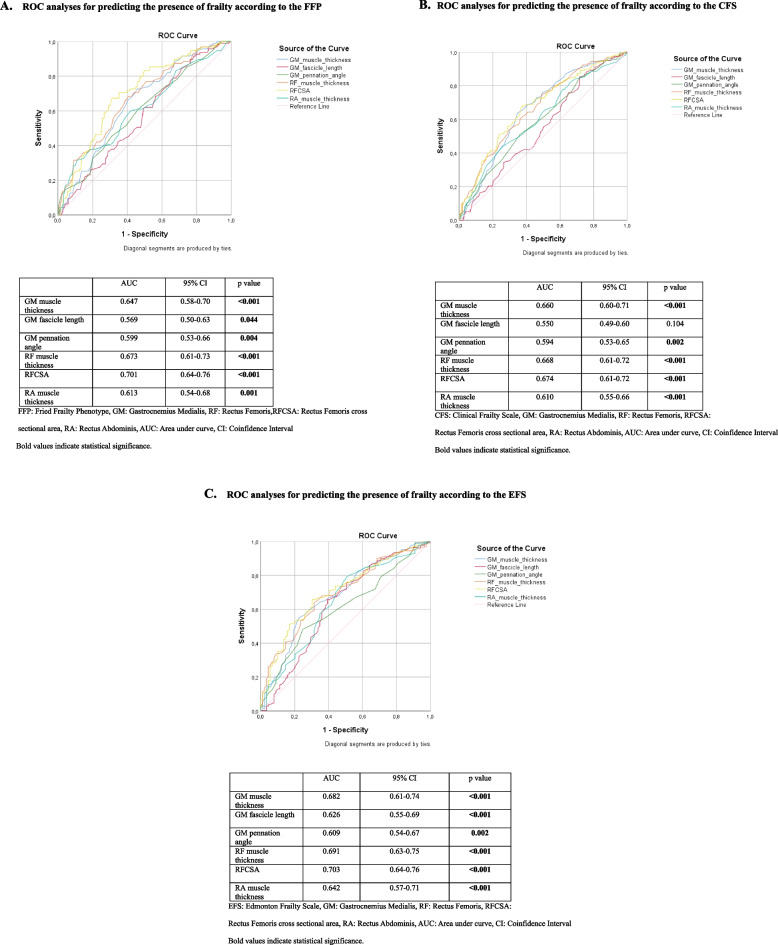
Area under the receiver operating characteristic curves for predicting frailty using the muscle ultrasound measurements. **A** ROC analyses for predicting the presence of frailty according to the FFP. **B** ROC analyses for predicting the presence of frailty according to the CFS. **C **ROC analyses for predicting the presence of frailty according to the EFS

In ROC analyses, GM muscle thickness, GM pennation angle, RF muscle thickness, RFCSA, and RA muscle thickness were significant for predicting the presence of frailty according to the FFP, CFS, and EFS. Only GM fascicle length was not significant for the CFS (Fig. 3A, B and C).

## Discussion

In this study, we showed that muscle thickness of GM, RF, and RA, fascicle length of GM, and pennation angle of GM and RFCSA were related to the FFP, CFS, and EFS scores. Particularly, GM pennation angle, RF muscle thickness, and RFCSA were associated with an increased risk of frailty. Besides muscle thickness of GM, RF, and RA, fascicle length of GM, pennation angle of GM, and RFCSA were significant for predicting the presence of frailty.

Frailty is the failure of homeostatic mechanisms by which many organs and systems are affected. Sarcopenia, characterized by a decrease in muscle strength and mass, and a decrease in physical performance is considered a key component of frailty [6]. Although the specific pathophysiological pathways underlying frailty are not clearly known, there is evidence that both malnutrition and sarcopenia may predispose to frailty, particularly in older adults [30]. In an individual living with frailty, muscle homeostasis is impaired, and rapid muscle loss occurs due to the neurologic, endocrine, and immune systems being affected. Therefore, although frailty and sarcopenia are separate geriatric syndromes, they are intertwined conditions. This vicious circle is reinforced by existing multimorbidities [31]. The presence of sarcopenia and frailty in a diabetic patient accelerates the mechanisms of insulin resistance, chronic inflammation, and mitochondrial dysfunction. The coexistence of these 3 conditions worsens the prognosis of DM in older patients, increases the frequency of micro-macrovascular complications, makes it difficult to reach treatment goals, and impairs treatment compliance [32]. Therefore, interventions to clarify the frailty-sarcopenia relationship in diabetic older adults are mandatory. In previous studies, the relationship between frailty and sarcopenia in diabetic patients was discussed mostly based on physical frailty [33, 34]. In our study, we demonstrated the relationship between muscle measurements and both physical, cumulative deficit and multidimensional frailty models.

In most of the studies trying to define the frailty-sarcopenia relationship with imaging techniques, CT is a widely used method, especially within oncology. In many cancer types, low muscle mass has been associated with adverse outcomes, poor prognosis, and mortality [35–37]. However, the results may differ in studies where frailty is also considered. Williams et al. showed that in 162 older adults with cancer, CT-measured muscle mass was poorly associated with frailty defined by the Carolina Frailty Index, but muscle density was more associated with frailty [38]. Another study found that sarcopenia was associated with chemotherapy toxicity, but not between sarcopenia and frailty, in older cancer patients evaluated by comprehensive geriatric assessment [39]. Controversy, Zwart et al. demonstrated the relationship between muscle mass measured by CT and frailty using the G8 frailty questionnaire and the Groningen Frailty Indicator, in older patients with head and neck cancer [40]. The relationship between sarcopenia and frailty has been tried to be demonstrated by different methods in different populations. Such that, Brown et al. showed this relationship in patients with aortic aneurysm using the Rockwood Clinical Frailty Scale and CT-derived muscle mass [41]. However, in the aforementioned studies, a single muscle group was evaluated with a single frailty scale. So that, in some studies, sarcopenia was evaluated as a frailty component without even using a frailty index, and CT–derived measurements as a frailty assessment tool, and sarcopenia-frailty-mortality relationship has been shown in patients who underwent transcatheter aortic valve replacement [42].

Muscle US is a current approach preferred in older adults to identify sarcopenia and correlates with many measurement methods and physical performance tests [43]. In a study of 136 hospitalized older adults, rectus femoris plus vastus intermedius thickness measured by the US was associated with frailty defined by the Frailty Index. And the authors suggested that US could be a component of a comprehensive geriatric assessment [44]. In another study conducted with the FFP, the relationship between vastus lateralis muscle thickness and frailty was shown in hospitalized older patients [45]. Mueller et al.‘s study in the intensive care unit showed the relationship between the rectus femoris cross-sectional area measured by the US and the Frailty Index, and they emphasized the importance of bedside US in risk stratification in critically ill patients [11]. In another study, it was emphasized that sarcopenia could be diagnosed with US in a diabetic older population living with frailty, but frailty indices and muscle evaluations were not compared [13]. In a study examining the relationship between multiple frailty definitions (Frailty Phenotype, Frailty Index, EFS, and CFS) and US, the relationship between bilateral anterior thigh thickness and the EFS was shown in hemodialysis recipients [12]. However, different results can be obtained in different studies conducted for the same purpose. Madden et al. showed that frailty detected by the Frailty Phenotype and CFS weakly correlated with vastus medialis muscle thickness in hospitalized older adults, and the authors suggested that studies on detecting frailty with a method such as US should be expanded [34]. However, most of these studies used a single frailty index and focused on a single muscle group, which is usually the anterior thigh muscle group. In this study, we evaluated muscle thickness, pennation angle, fascicle length, and cross-sectional area, which are muscle parameters recommended by the SARCUS working group [8]. We showed the correlation between GM, RF, RA muscle thickness, GM fascicle length, GM pennation angle, RFCSA, and the FFP, CFS, and EFS scores in a diabetic older population. All these parameters were associated with the FFP, CFS, and EFS scores on univariable analyses, and retained association for GM pennation angle, RF muscle thickness, and RFCSA on the fully adjusted model. The significant results of all muscle parameters in ROC analyses showed that these parameters can be used in the estimation of frailty diagnosis. To the best of our knowledge, this is the first study that evaluates the relationship between GM muscle thickness, GM pennation angle, GM fascicle length, RA muscle thickness, RFCSA, and RA muscle thickness obtained by the US with the FFP, CFS, and EFS in a diabetic geriatric population. The selection of ultrasound measurement parameters for frailty assessment in DM patients should consider practical aspects such as feasibility and patient burden. While more complex ultrasound parameters may provide valuable insights into muscle health, they may also require specialized equipment and expertise, potentially increasing the burden on patients and healthcare providers. Therefore, a balance between clinical utility and practical considerations is crucial when determining the most appropriate ultrasound measurement parameter for frailty assessment in DM patients. According to the results of correlation and regression analyses of this study, the strongest relationship between frailty and ultrasound parameters was observed with rectus femoris (RF) muscle thickness and RF cross-sectional area (CSA), therefore could be more appropriate for identifying frailty in older patients with DM.

Strengths of our study include a sufficient number of patients to examine our hypothesis, a study population consisting of diabetic patients, evaluation of multiple frailty definitions, evaluation of more than one muscle group with more than one parameter, and the selection of participants from community-dwelling older adults.

The study’s limitations include its cross-sectional design, which precludes the establishment of causality. Additionally, muscle echo intensity was not performed owing to the absence of the equipment and software capabilities for such measurements in our ultrasound. Lastly, in evaluating the intraobserver reliability, we were unable to perform repeated measurements for pennation angle and fascicle length. Future research should aim to explore the relationship between frailty and sarcopenia through prospective studies, considering frailty as a dynamic process.

## Conclusions

In conclusion, US-derived regional muscle measurements were associated with different frailty definitions in both physical, cumulative, and multidimensional models in a diabetic geriatric population. This finding suggests that muscle US parameters may be useful in other models, not only to identify physical frailty.

### Supplementary Information

**Supplementary Material 1.**

## Data Availability

The datasets used and/or analysed during the current study are available from the corresponding author on reasonable request.

## Abbreviations

US: Ultrasound
FFP: Fried Frailty Phenotype
CFS: Clinical Frailty Scale
EFS: Edmonton Frailty Scale
GM: Gastrocnemius Medialis
RF: Rectus Femoris
RFCSA: Rectus Femoris cross-sectional area
RA: Rectus Abdominis
EO: External Oblique
IO: Internal Oblique
TA: Transverse Abdominis
DM: Diabetes Mellitus
CGA: Comprehensive Geriatric Assessment
ADL: Activities of Daily Living
IADL: Instrumental Activities of Daily Living
MMSE: Mini-Mental State Examination
MNA-sf: Mini-Nutritional Assessment-short form
YGDS: Yesavage Geriatric Depression Scale
BMI: Body Mass Index
