# Supplementary material for: Role of muscle ultrasound in frailty assessment in older adults with type 2 diabetes mellitus
Source: BMC Geriatr. 2024 May 4;24:397. doi: 10.1186/s12877-024-05008-y (PMC11069196; doi:10.1186/s12877-024-05008-y)
Supplement: Supplementary file 1 — Supplementary Material 1. [file 12877_2024_5008_MOESM1_ESM.docx]

**Table S1. Correlations between muscle measurements and frailty definitions among male older adults with diabetes mellitus**

|  | GM muscle thickness | GM fascicle length | GM pennation angle | RF  muscle thickness | RFCSA | RA muscle thickness | EO muscle thickness | IO  muscle thickness | TA  muscle thickness |
| --- | --- | --- | --- | --- | --- | --- | --- | --- | --- |
| FFP | r: -0.270  p<:0.002 | r: -0.026  p:0.76 | r: -0.196  p=0.025 | r: -0.314  p<0.001 | r: -0.328  p<0.001 | r: -0.212  p:0.015 | r: -0.013  p: 0.88 | r: -0.06  p: 0.49 | r: -0.054  p: 0.54 |
| CFS | r: -0.240  p:0.006 | r: -0.064  p:0.47 | r: -0.198  p:0.02 | r: -0.221  p:0.01 | r: -0.264  p:0.02 | r: -0.182  p:0.037 | r: -0.023  p: 0.79 | r: -0.129  p: 0.14 | r:- 0.021  p: 0.81 |
| EFS | r: -0.225  p:0.01 | r: -0.041  p:0.64 | r: -0.131  p:0.14 | r: -0.149  p:0.09 | r: -0.206  p:0.018 | r: -0.123  p:0.16 | r: -0.019  p: 0.83 | r: -0.079  p: 0.37 | r: 0.010  p: 0.91 |

**Table S2. Correlations between muscle measurements and frailty definitions among female older adults with diabetes mellitus**

|  | GM muscle thickness | GM fascicle length | GM pennation angle | RF  muscle thickness | RFCSA | RA muscle thickness | EO muscle thickness | IO  muscle thickness | TA  muscle thickness |
| --- | --- | --- | --- | --- | --- | --- | --- | --- | --- |
| FFP | r: -0.342  p<0.001 | r: -0.167  p=0.001 | r: -0.164  p=0.002 | r: -0.372  p<0.001 | r: -0.408  p<0.001 | r: -0.238  p<0.001 | r: -0.040  p: 0.446 | r: -0.107  p: 0.040 | r: 0.015  p: 0.776 |
| CFS | r: -0.363  p<0.001 | r: -0.180  p<0.001 | r: -0.181  p<0.001 | r: -0.360  p<0.001 | r: -0.366  p<0.001 | r: -0.237  p<0.001 | r: -0.033  p: 0.529 | r: -0.088  p: 0.091 | r: 0.049  p: 0.353 |
| EFS | r: -0.396  p<0.001 | r: -0.251  p<0.001 | r: -0.204  p<0.001 | r: -0.323  p<0.001 | r: -0.371  p<0.001 | r: -0.257  p<0.001 | r: -0.083  p: 0.114 | r: -0.126  p: 0.016 | r: 0.061  p: 0.243 |

**Figure S1. ROC analyses for predicting the presence of frailty according to the FFP for male older adults with diabetes mellitus**

**
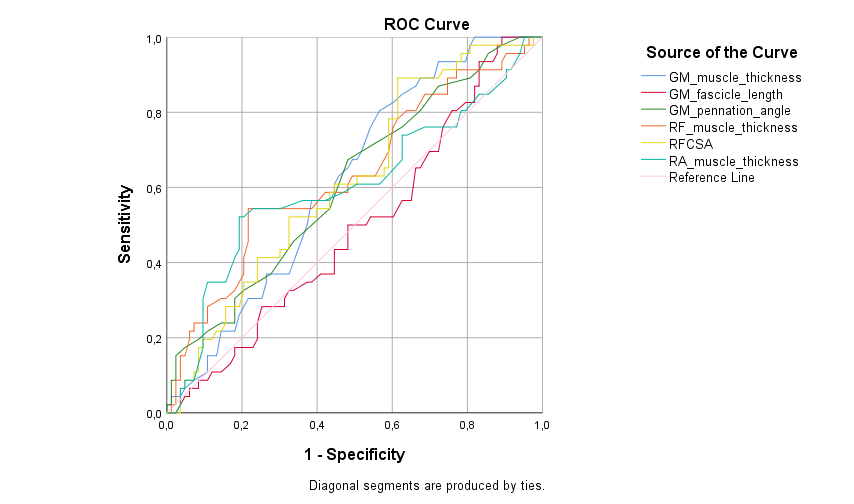
**

|  | AUC | 95% CI | p value |
| --- | --- | --- | --- |
| GM muscle thickness | 0.619 | 0.52-0.71 | 0.025 |
| GM fascicle length | 0.495 | 0.39-0.59 | 0.926 |
| GM pennation angle | 0.612 | 0.51-0.71 | 0.035 |
| RF muscle thickness | 0.633 | 0.53-0.73 | 0.012 |
| RFCSA | 0.618 | 0.52-0.71 | 0.027 |
| RA muscle thickness | 0.606 | 0.49-0.71 | 0.047 |

FFP: Fried Frailty Phenotype, GM: Gastrocnemius Medialis, RF: Rectus Femoris,RFCSA: Rectus Femoris cross

sectional area, RA: Rectus Abdominis, AUC: Area under curve, CI: Coinfidence Interval

**Figure S2. ROC analyses for predicting the presence of frailty according to the CFS for male older adults with diabetes mellitus**

**
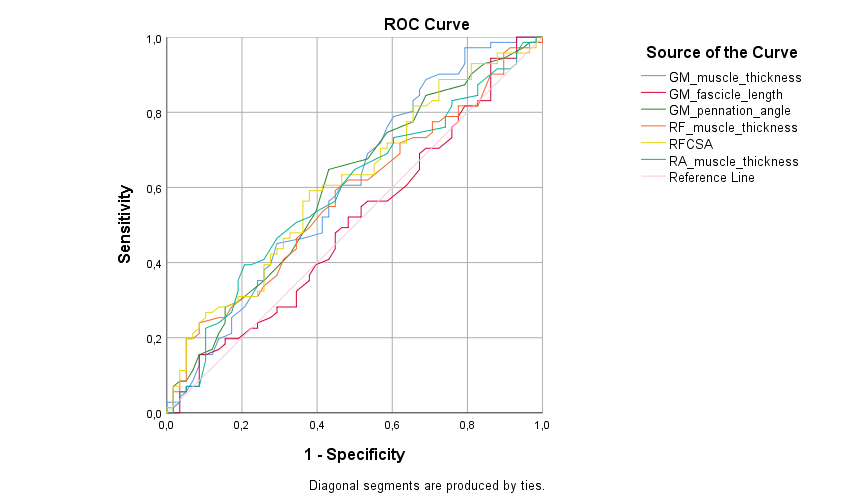
**

|  | AUC | 95% CI | p value |
| --- | --- | --- | --- |
| GM muscle thickness | 0.606 | 0.50-0.70 | 0.038 |
| GM fascicle length | 0.504 | 0.40-0.60 | 0.938 |
| GM pennation angle | 0.602 | 0.50-0.70 | 0.046 |
| RF muscle thickness | 0.574 | 0.47-0.67 | 0.147 |
| RFCSA | 0.611 | 0.51-0.70 | 0.031 |
| RA muscle thickness | 0.588 | 0.48-0.68 | 0.087 |

CFS: Clinical Frailty Scale, GM: Gastrocnemius Medialis, RF: Rectus Femoris,RFCSA: Rectus Femoris cross

sectional area, RA: Rectus Abdominis, AUC: Area under curve, CI: Coinfidence Interval

**Figure S3. ROC analyses for predicting the presence of frailty according to the EFS for male older adults with diabetes mellitus**

**
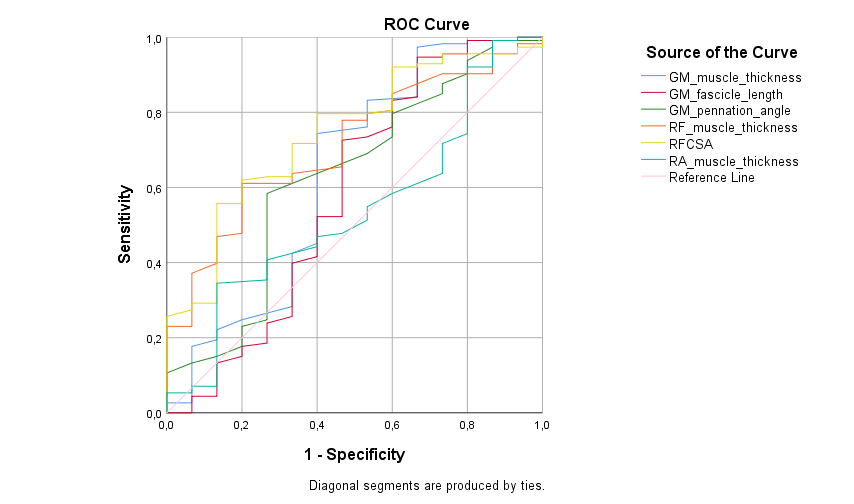
**

|  | AUC | 95% CI | p value |
| --- | --- | --- | --- |
| GM muscle thickness | 0.635 | 0.45-0.81 | 0.090 |
| GM fascicle length | 0.583 | 0.39-0.77 | 0.298 |
| GM pennation angle | 0.627 | 0.46-0.78 | 0.112 |
| RF muscle thickness | 0.712 | 0.58-0.83 | 0.008 |
| RFCSA | 0.745 | 0.62-0.86 | 0.002 |
| RA muscle thickness | 0.538 | 0.38-0.69 | 0.633 |

EFS: Edmonton Frailty Scale, GM: Gastrocnemius Medialis, RF: Rectus Femoris,RFCSA: Rectus Femoris cross

sectional area, RA: Rectus Abdominis, AUC: Area under curve, CI: Coinfidence Interval

**Figure S4. ROC analyses for predicting the presence of frailty according to the FFP for female older adults with diabetes mellitus**

**
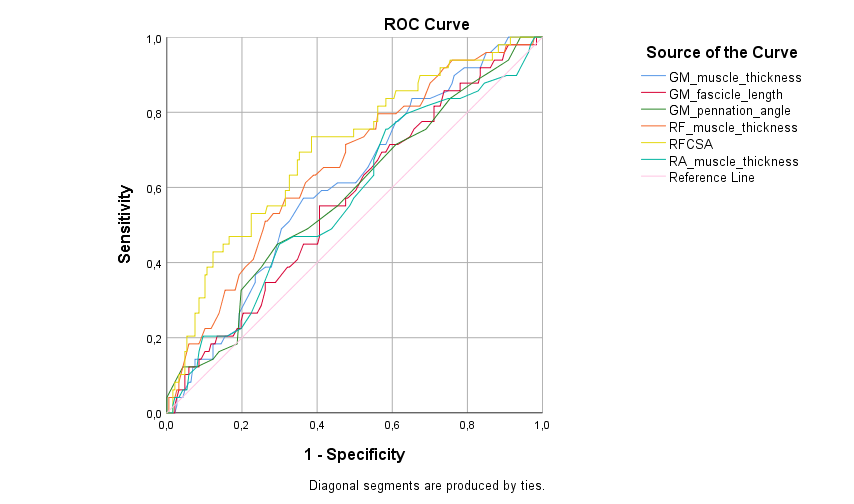
**

|  | AUC | 95% CI | p value |
| --- | --- | --- | --- |
| GM muscle thickness | 0.609 | 0.52-0.69 | 0.019 |
| GM fascicle length | 0.569 | 0.48-0.65 | 0.137 |
| GM pennation angle | 0.580 | 0.49-0.66 | 0.087 |
| RF muscle thickness | 0.658 | 0.57-0.74 | 0.043 |
| RFCSA | 0.702 | 0.62-0.78 | <0.001 |
| RA muscle thickness | 0.571 | 0.48-0.66 | 0.127 |

FFP: Fried Frailty Phenotype, GM: Gastrocnemius Medialis, RF: Rectus Femoris,RFCSA: Rectus Femoris cross

sectional area, RA: Rectus Abdominis, AUC: Area under curve, CI: Coinfidence Interval

**Figure S5. ROC analyses for predicting the presence of frailty according to the CFS for female older adults with diabetes mellitus**

**
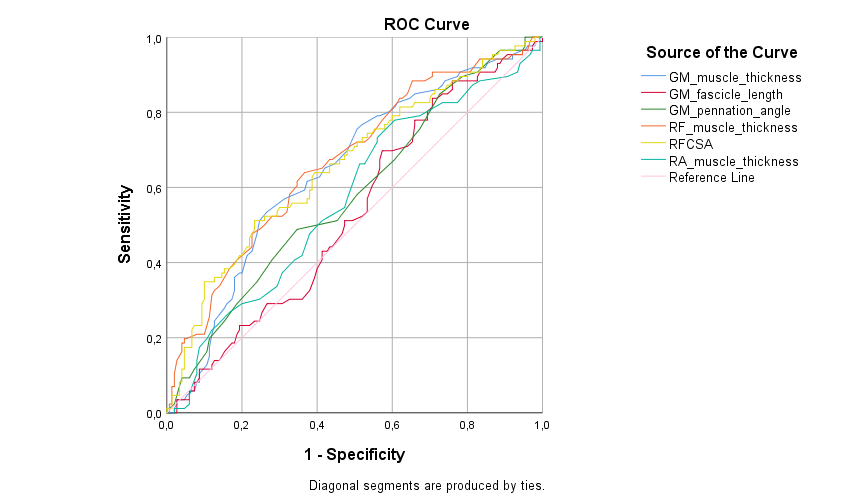
**

|  | AUC | 95% CI | p value |
| --- | --- | --- | --- |
| GM muscle thickness | 0.647 | 0.57-0.72 | <0.001 |
| GM fascicle length | 0.532 | 0.45-0.60 | 0.410 |
| GM pennation angle | 0.582 | 0.50-0.65 | 0.037 |
| RF muscle thickness | 0.669 | 0.59-0.74 | <0.001 |
| RFCSA | 0.660 | 0.58-0.73 | <0.001 |
| RA muscle thickness | 0.572 | 0.49-0.64 | 0.066 |

CFS: Clinical Frailty Scale, GM: Gastrocnemius Medialis, RF: Rectus Femoris,RFCSA: Rectus Femoris cross

sectional area, RA: Rectus Abdominis, AUC: Area under curve, CI: Coinfidence Interval

**Figure S6. ROC analyses for predicting the presence of frailty according to the EFS for female older adults with diabetes mellitus**

**
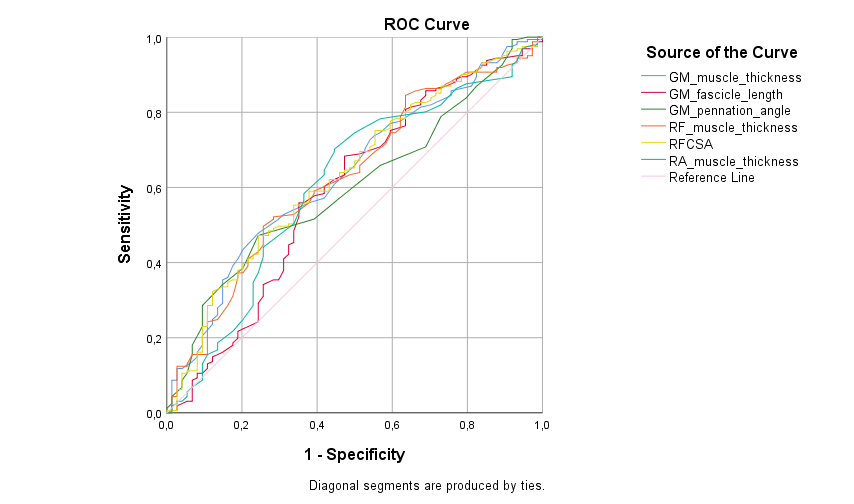
**

|  | AUC | 95% CI | p value |
| --- | --- | --- | --- |
| GM muscle thickness | 0.634 | 0.55-0.70 | 0.001 |
| GM fascicle length | 0.591 | 0.50-0.67 | 0.025 |
| GM pennation angle | 0.600 | 0.52-0.67 | 0.014 |
| RF muscle thickness | 0.626 | 0.55-0.70 | 0.002 |
| RFCSA | 0.633 | 0.55-0.70 | 0.001 |
| RA muscle thickness | 0.610 | 0.53-0.68 | 0.007 |

EFS: Edmonton Frailty Scale, GM: Gastrocnemius Medialis, RF: Rectus Femoris,RFCSA: Rectus Femoris cross

sectional area, RA: Rectus Abdominis, AUC: Area under curve, CI: Coinfidence Interval
